# The pipeline for drugs for control and elimination of neglected tropical diseases: 2. Oral anti-infective drugs and drug combinations for off-label use

**DOI:** 10.1186/s13071-023-05909-8

**Published:** 2023-10-31

**Authors:** Kenneth M. Pfarr, Anna K. Krome, Issraa Al-Obaidi, Hannah Batchelor, Michel Vaillant, Achim Hoerauf, Nicholas O. Opoku, Annette C. Kuesel

**Affiliations:** 1 Institute of Medical Microbiology, Immunology and Parasitology, University Hospital Bonn, Bonn, Germany; 2 German Center for Infection Research (DZIF), Partner Site Bonn-Cologne, Bonn, Germany; 3 Department of Pharmaceutical Technology and Biopharmaceutics, University of Bonn, Bonn, Germany; 4 Strathclyde Institute of Pharmacy and Biomedical Sciences, University of Strathclyde, Glasgow, UK; 5 Competence Center for Methodology and Statistics, Luxembourg Institute of Health, Strassen, Grand Duchy of Luxembourg; 6 Department of Epidemiology and Biostatistics School of Public Health, University of Health and Allied Sciences, Hohoe, Ghana; 7 UNICEF/UNDP/World Bank/WHO Special Programme for Research and Training in Tropical Diseases (WHO/TDR), World Health Organization, Geneva, Switzerland

**Keywords:** Neglected topical diseases, Drug repurposing, Drug reprofiling, Off-label use, Acedapsone, Albendazole, Amoxicillin, Artemether, Artemisinins, Artesunate, Arterolane, Azithromycin, Azoles, Balapiravir, Celgosivir, Chloroquine, Clarithromycin, Clavulanate, Disulfiram, Dapsone, Diethylcarbamazine, Disulfiram, Doxycycline, Fluconazole, Imatinib, Ivermectin, Isavuconazonium, Itraconazole, Ketoconazole, Levamisole, Lumefantrine, Mebendazole, Mefloquine, Moxidectin, Moxifloxacin, Nitazoxanide, Oxamniquine, Oxantel, Piperaquine, Posaconazole, Praziquantel, Pyrantel pamoate, Ravuconazole, Ribavirin, Rifampicin, Rifapentine, Sparfloxacin, Streptomycin, Tribendimidine, UV-4B, CURE ID

## Abstract

In its ‘Road map for neglected tropical diseases 2021–2030’, the World Health Organization outlined its targets for control and elimination of neglected tropical diseases (NTDs) and research needed to achieve them. For many NTDs, this includes research for new treatment options for case management and/or preventive chemotherapy. Our review of small-molecule anti-infective drugs recently approved by a stringent regulatory authority (SRA) or in at least Phase 2 clinical development for regulatory approval showed that this pipeline cannot deliver all new treatments needed. WHO guidelines and country policies show that drugs may be recommended for control and elimination for NTDs for which they are not SRA approved (i.e. for ‘off-label’ use) if efficacy and safety data for the relevant NTD are considered sufficient by WHO and country authorities. Here, we are providing an overview of clinical research in the past 10 years evaluating the anti-infective efficacy of oral small-molecule drugs for NTD(s) for which they are neither SRA approved, nor included in current WHO strategies nor, considering the research sponsors, likely to be registered with a SRA for that NTD, if found to be effective and safe. No such research has been done for yaws, guinea worm, *Trypanosoma brucei gambiense* human African trypanosomiasis (HAT), rabies, trachoma, visceral leishmaniasis, mycetoma, *T. b. rhodesiense* HAT, echinococcosis, taeniasis/cysticercosis or scabies. Oral drugs evaluated include sparfloxacin and acedapsone for leprosy; rifampicin, rifapentin and moxifloxacin for onchocerciasis; imatinib and levamisole for loiasis; itraconazole, fluconazole, ketoconazole, posaconazole, ravuconazole and disulfiram for Chagas disease, doxycycline and rifampicin for lymphatic filariasis; arterolane, piperaquine, artesunate, artemether, lumefantrine and mefloquine for schistosomiasis; ivermectin, tribendimidine, pyrantel, oxantel and nitazoxanide for soil-transmitted helminths including strongyloidiasis; chloroquine, ivermectin, balapiravir, ribavirin, celgosivir, UV-4B, ivermectin and doxycycline for dengue; streptomycin, amoxicillin, clavulanate for Buruli ulcer; fluconazole and isavuconazonium for mycoses; clarithromycin and dapsone for cutaneous leishmaniasis; and tribendimidine, albendazole, mebendazole and nitazoxanide for foodborne trematodiasis. Additional paths to identification of new treatment options are needed. One promising path is exploitation of the worldwide experience with ‘off-label’ treatment of diseases with insufficient treatment options as pursued by the ‘CURE ID’ initiative.

**Graphical abstract:**

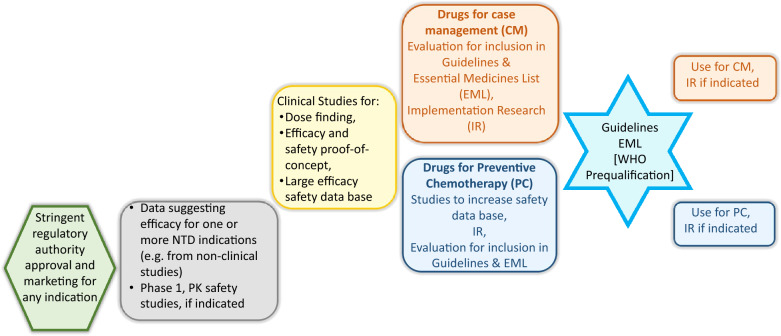

**Supplementary Information:**

The online version contains supplementary material available at 10.1186/s13071-023-05909-8.

## Background

Around 1 billion people, primarily in low- and middle-income countries (LMIC), are affected by Neglected Tropical Diseases (NTDs) [1]. In 2020, WHO provided an overview of current strategic interventions and new tools and approaches needed to achieve the targets laid out in its 2021–2030 roadmap for NTDs (the ‘Roadmap’) [1]. New tools needed include, but are not limited to, new drugs, optimized drug regimens and/or new formulations.

We have previously provided an overview of new small-molecule anti-infective molecular entities (drugs) in the pipeline for NTD control and elimination strategies which have recently been approved by a regulatory authority for treatment of an NTD or are in at least Phase 2 clinical development for regulatory approval for an NTD [2]. Research and development (R&D) of new drugs comes with a significant risk of failure and it takes many years even from initiation of Phase 2 clinical development to the first regulatory agency approval (registration). R&D for new drugs also requires significant financial resources to achieve regulatory registration and further resources to maintain the registration [3]. Furthermore, studies beyond those that supported regulatory approval may be needed to provide the basis for inclusion of the new drug in WHO guidelines, country policies and practice [2].

Repurposing (also referred to as ‘reprofiling’ or ‘repositioning’) drugs already registered provides a cheaper, less risky and faster path to new treatment options. Repurposing of registered veterinary drugs for human use led to a number of drugs used today for NTDs [2]. Repurposing of drugs registered for one indication in humans for another indication can provide an even cheaper, less risky and faster pathway to new treatment options: the pre-clinical toxicology profile is known, a formulation for human use is available, the pharmacokinetics (PK) and safety of the drug in healthy volunteers and the potential for drug-drug interactions have been characterized and data are available on the safety profile of the drug in the approved indication(s) with the approved formulation(s) and dosing regimen(s). Therefore, repurposing drugs approved for indication(s) in humans can, in most cases, be initiated with Phase 2 studies with the available formulation to obtain proof of concept for efficacy in the new indication and identify a dose regimen to be evaluated in Phase 3 studies. The Phase 3 studies will be designed to establish the efficacy with acceptable statistical significance and to better characterize the safety profile of the chosen regimen in the targeted indication. Depending on the intended use context, these studies may have to be followed by additional larger scale studies to support guidelines and policies and by implementation research as is often necessary for new drugs [2].

Availability of efficacy and safety data for an indication may qualify the drug for inclusion in guidelines and policies even if the drug is not registered for the new indication, i.e. will be used ‘off-label’. Some WHO guidelines for ‘Preventive Chemotherapy’ (PC, i.e. drug administration to specified populations irrespective of the presence of symptoms or infection; for an overview of WHO guidelines see supplementary information in [2]) include drugs and drug combinations which have not been registered for the disease for which WHO recommends their use during PC. An example is PC for elimination of LF as a public health problem: in onchocerciasis co-endemic countries albendazole combined with ivermectin is recommended, in countries where onchocerciasis is not co-endemic albendazole combined with diethylcarbamazine or with diethylcarbamazine and ivermectin is recommended and in *Loa loa* co-endemic areas PC with albendazole alone is recommended [1, 2, 4, 5]. The basis for the introduction of albendazole + ivermectin and albendazole + diethylcarbamazine for PC of LF was studies on the effect of ivermectin on LF microfilaraemia as well as large-scale studies on the safety of the combination treatments conducted to support a potential WHO guideline as per WHO expert consultations [6–8]. LF is an indication for ivermectin (under the brand name Mectizan) in the prescribing information (label) authorized by the French Regulatory Authority (Agence nationale de sécurité du médicament et des produits de santé) [9] but not in the prescribing information authorized for ivermectin (under the brandname Stromectol) by the US FDA. To our knowledge, indications for albendazole by national regulatory authorities may include strongyloidiasis, ascariasis, trichuriasis and infections with *Echinococcus granulosus*, *Enterobius vermicularis*, *Giardia intestinalis*, *G. duodenalis*, *Taenia solium*, *T. saginata*, *Trichinella spiralis* or hookworm infections but not LF. LF was recently included among ivermectin and albendazole indications for products prequalified by the WHO Prequalification Programme [10, 11]. For another ivermectin product, prequalified via the abbreviated prequalification process for products approved by stringent regulatory authorities (SRA [12]) in this case the US FDA [13, 14]), the prescribing information is that approved by the US FDA, i.e. does not include LF. However, the WHO Prequalification Programme specifies in Part 1 of the documentation that WHO recommended uses include LF, scabies, strongyloidiasis and, when administered with albendazole, soil-transmitted intestinal worm infections [15].

Here, we provide an overview of clinical studies for repurposing small-molecule anti-infective drugs, as mono- or combination treatment, for an NTD. This research is driven primarily by two concerns: (1) potential development of resistance when current strategies are based on the use of a single drug and (2) inadequate efficacy or safety of available drugs/drug combinations. Both could be addressed by new treatment options with repurposed drugs including combination treatment.

We included clinical research which meets the following criteria:

- The drugs evaluated are approved for human use and administered orally;
- The drugs have not received regulatory approval for the NTD for which they are being clinically evaluated and/or are not included in current NTD control and elimination strategies as per the Roadmap for the NTD for which their efficacy was evaluated [1, 2];
- Based on the study sponsors, submission of an application for regulatory registration for the NTD should the research show that the drug is efficacious and safe for the new NTD indication appears unlikely, i.e. potential use would be off-label;
- The anti-infective effect was evaluated.

We did not consider co-administration of different drugs in view of integration of current strategies for different diseases as ‘combination treatment’; consequently, relevant studies are not included. Unless previously provided by us [2] or in recent reviews by others, we are summarizing basic information on the drugs, prior research that motivated repurposing and the main conclusions to date.

For information on regulatory approval, we searched first for registration in the US for the following reasons: (1) given the characteristics of the US market, the probability of registration for indications present in the US population is highest, and even drugs for indications not endemic in the US may be registered in the US (e.g. ivermectin for onchocerciasis), (2) the US FDA website provides an excellent, easy-to-search data base on approved drugs [16]. The data base includes current US FDA approved labelling (prescribing information), past labelling and for many drugs the very informative assessment of the US FDA reviewers at the time of the original registration. When needed, we also searched the websites of other SRAs that provide detailed product information with reviewer assessment (European Medicines Agency, the United Kingdom Medicines and Healthcare products Regulatory Agency). Search of the websites of other SRAs (Ministry of Health, Labour and Welfare of Japan, Swissmedic, Health Canada, Australia, Iceland, Liechtenstein, Norway [12]) was not necessary.

Clinical studies were identified through search of the WHO International Clinical Trials Registry Platform (ICTRP) for studies registered in a clinical trials platform in the past approximately 10 years. Additional file 1: Table S1 shows the ICTRP source registries and last data file import date into the ICTRP as of the search date of 8 February 2023. For studies for which the literature search identified no publications, we accessed the source registry file for any update on the study status. We excluded studies with records that had not been updated for 2 years after the study completion date (as provided in the record or estimated based on recruitment start and study duration) or for 3 years if the information in the record was insufficient to estimate the study completion date. Studies completed as per the source registry but for which we could not identify a publication as well as ongoing and planned studies are included in Table 1. Table 2 provides an overview of the drugs meeting our inclusion for this manuscript combined with those we reviewed previously [2] together with the drugs in current WHO-recommended strategies and the needs for improved treatment options WHO identified in the Roadmap.

**Table 1:** Planned, ongoing or unpublished completed studies in the ICTRP as of 8 February 2023 evaluating small-molecule anti-infective drugs approved for human use but not included in current WHO recommended NTD strategies

WHO target NTD Source registry trial ID	Scientific title	Country of conduct	Registration date^a^ Status^b^
Elimination			
Onchocerciasis			
NCT02078024 ISRCTN50035143	Comparison of ivermectin alone with albendazole (ALB) plus ivermectin (IVM) in their efficacy against onchocerciasis in the Volta Region, Ghana	Ghana	20140228 (completed)
NCT04188301 PACTR 201906665550709	Safety and efficacy of combination therapy with ivermectin, diethylcarbamazine, and albendazole (IDA) for individuals with onchocerciasis	Ghana	20191202 20190624 Active, not recruiting
ISRCTN43697583	The efficacy of rifapentine 900 mg/d plus moxifloxacin 400 mg/d given for 14 or 7 days against onchocerciasis: a randomized, parallel-group, open-label, phase II pilot trial	Ghana	20150417 (completed)
PACTR202212910949177	Exploiting the synergy of registered drugs rifampicin and albendazole to shorten the treatment duration of macrofilaricide for the cure of onchocerciasis in areas co-endemic with loiasis: an exploratory pilot phase II clinical trial study	Cameroon	20221219 (not yet recruiting, last follow-up December 2025)
ISRCTN38954299	Assessment of the efficacy of 7-14 days treatment with either high dose rifampicin 35 mg/kg/day plus albendazole (400 mg/day), rifampicin 10 mg/kg/day plus albendazole (400 mg/d) or doxycycline 200 mg/day plus albendazole (400 mg/day) in the treatment of onchocerciasis: a randomized, controlled, parallel-group, open-label, phase II pilot trial Cameroon	Cameroon	20210604 Active, not recruiting, last follow-up May 2023)
PACTR 202009704006025	The efficacy of rifampicin 35mg/kg/d plus albendazole 400mg/d given for 7 or 14 days against lymphatic filariasis and onchocerciasis: a randomized, controlled, parallel-group, open-label, phase II pilot trial	Ghana	20200713 Recruiting (last follow-up Dec. 2024)
Loiasis			
ISRCTN14889921	Pre-treatment of loiasis caused by the parasitic African eye worm *Loa loa* in Gabon with the antiparasitic medication albendazole among patients with a high risk of adverse events after another antiparasitic administration, ivermectin	Gabon	20221129 (retrospectively) Projected completion February 2023
PACTR201807197019027	Safety and efficacy of different albendazole-based treatment regimens to reduce microfilaraemia in subjects infected by *Loa loa* in an endemic area of Gabon: a randomised controlled open-label pilot study [3 weeks albendazole 400mg bid vs. 3 weeks albendazole followed by 2 weeks albendazole vs. 3 weeks albendazole followed by a single dose of 150 µg/kg ivermectin as soon as microfilarial loads have dropped to < 4000 mf/ml in > 90% of subjects vs. no treatment	Gabon	20180607 (retrospectively) Completed
Elimination as public health problem			
Chagas disease			
Registro Brasileira de Ensaios Clinicos ID: RBR-5n4htp	Disulfiram repurposing in the combined chemotherapy of Chagas disease—phase I/II clinical trial	Brazil	20200217 Recruiting
Lymphatic filariasis			
PACTR202009704006025	The efficacy of rifampicin 35mg/kg/d plus albendazole 400mg/d given for 7 or 14 days against lymphatic filariasis and onchocerciasis a randomized, controlled, parallel-group, open-label, phase II pilot trial	Ghana	20200713 Recruiting (last follow-up Dec. 2024)
ISRCTN15320064	Comparing the effectiveness of test and treat approaches with doxycycline or the triple-drug therapy with ivermectin/diethylcarbamazine/albendazole vs. ivermectin/albendazole for targeted elimination of lymphatic filariasis in a phase III clinical trial	Ghana	20220,329 Not yet recruiting (last update August 2022)
Schistosomiasis			
NCT03893097	A proof-of-concept trial to evaluate artesunate-mefloquine as a novel alternative treatment for schistosomiasis in African children	Senegal	20190328 Completed
STH including strongyloidiasis			
PACTR202011642671155	An adaptive phase II/III single-blinded, randomized, multi-centre, parallel-group, active-controlled, superiority study to evaluate the safety and efficacy of a single day or 3-day single dose of an albendazole-ivermectin coformulation vs. albendazole for the treatment of soil-transmitted helminth infections (*Trichuris trichiura*, hookworm, *Strongyloides stercoralis*) in paediatric and young adult population	Kenya, Mozambique, Ethiopia	20201015 Recruiting Last follow-up 31 July 2023
Control			
Dengue			
NCT03432442	Pharmacokinetics and pharmacodynamics of ivermectin in pediatric dengue patients	Thailand	20180214 Completed (last update February 2021)
CTRI/2021/09/036661	Role of doxycycline in the treatment of dengue infection	India	20210920 Not yet recruiting (last update September 2021)
Buruli ulcer			
PACTR202209521256638	Shortening Buruli ulcer treatment: WHO recommended vs. a novel beta-lactam-containing therapy—phase III evaluation in West Africa [rifampicin plus clarithromycin (RC) for 8 weeks vs. RC plus amoxicillin/clavulanate for 4 weeks]	Ghana, Côte d’Ivoire, Togo	20220912 Planned start December 2022, planned final follow-up November 2023 Not yet recruiting
NCT05169554	Beta-lactam containing regimen for the shortening of Buruli ulcer disease therapy: comparison of 8 weeks standard therapy (rifampicin plus clarithromycin) vs. 4 weeks standard plus amoxicillin/clavulanate therapy (RC8 vs. RCA4)	Benin	20211227 Recruiting, Primary outcome data November 2024
Cutaneous leishmaniasis			
IRCT20140211016554N4	Evaluation of the effectiveness of oral dapsone with intralesional antimoniate in treatment of cutaneous leishmaniasis in comparison with intralesional antimoniate alone	Islamic Republic of Iran	20190428 Completed

^a^Registration date provided as year month day. ^b^ Status as of source registry, accuracy of the status information depends on the timeliness with which sponsors update the source registry. If a study was registered in more than one registry, the most up-to-date status information at the time of writing of the manuscript is provided

*CTRI* Clinical Trials Registry India, *ICTRP* WHO International Clinical Trials Registry Platform, *ISRCTN* registry managed by BMC, Springer Nature, *NCT* Clinical Trials.gov, *PACTR* Pan African Clinical Trial Registry, *RBR* Registro Brasileiro de Ensaios Clinicos

**Table 2:** Overview of small-molecule anti-infective drugs evaluated alone or in combination in the past 10 years for NTDs for which they are not registered or included in current WHO NTD strategies

WHO objective NTD [Drugs included in WHO strategies] [1, 2]	Gaps in/actions required for anti-infective drugs as per 2030 roadmap [1, 2]	1.In at least Phase 2 development for regulatory approval or recently approved [2] 2.Evaluated in the past 10 years for off-label use as per ICTRP records
Eradication		
Yaws [azithromycin, benzathine benzylpenicillin]	–	1.None 2.None
Guinea worm [–]	Drugs for case management	1.None 2.None
Elimination of transmission		
Leprosy [rifampicin, dapsone, clofazimine, clarithromycin, minocycline or a quinolone (ofloxacin, levofloxacin or moxifloxacin)	New drugs or combinations	1.Bedaquiline 2.Sparfloxacin, acedapsone [21]
Human African trypanosomiasis (*gambiense) *[fexinidazole, eflornithine-nifurtimox, pentamidine]	Safe and efficient single oral dose for both stages to help integration of treatment into primary health system Oral formulation for children < 6 years	1.Acoziborole 2.None
Onchocerciasis [ivermectin]	Macrofilaricide Efficacy and safety of moxidectin in children and community settings Safe drugs safe in *Loa loa* co-endemic areas	1.Moxidectin, emodepside, flubentylosin (TylAMac, ABBV-4083), oxantel pamoate, Oxfendazole 2.Albendazole in combination with ivermectin, albendazole and diethylcarbamazine in combination with ivermectin, antibiotics (rifampicin, rifapentin, moxifloxacin); Loiasis focussed: imatinib (discontinued), levamisole
Elimination as a public health problem		
Rabies [none]	Monoclonal antibodies Anti-virals and agents promoting entry of drugs, antibodies and immune effectors cells across the blood-brain barrier	1.None 2.None
Trachoma [azithromycin, tetracycline]	–	1.None 2.None
Chagas disease [benznidazole, nifurtimox]	Dosage and duration of benznidazole and nifurtimox treatment Combination treatment, new drugs	1.Fexinidazole, fosravuconazole (discontinued) 2.Azoles (itraconazole, fluconazole, ketoconazole, posaconazole and ravuconazole) disulfiram
Human African Trypanosomiasis (*rhodesiense) *[suramin, melarsoprol]	Safe, efficient treatments to replace toxic arsenic-based melarsoprol	1.Fexinidazole 2.None
Visceral leishmaniasis [pentavalent antimonials, liposomal amphotericin B, paromomycin, miltefosine]	New, safe, cheap oral drugs not requiring cold chain Shorter first line regimens in East Africa More treatment options including combination treatments to mitigate risk of resistance	1.LXE408 2.None
Lymphatic filariasis [albendazole, ivermectin, diethylcarbamazine]	Macrofilaricide, drug safe in *Loa loa*-infected individuals	1.Moxidectin 2.Antibiotics: doxycycline [22], rifampicin
Schistosomiasis [praziquantel]	Improved praziquantel and pediatric formulation New drugs to complement praziquantel in case of resistance	1.None 2.Antimalarial drugs (arterolane, piperaquine, artesunate, artemether, lumefantrine, mefloquine)
Soil transmitted helminths including strongyloidiasis [albendazole, mebendazole]	More effective medicines and drug combinations against *T. trichiura* and hookworm infections Drugs and drug combinations to be used in case of emergence of drug resistance	1.Moxidectin, emodepside, oxantel pamoate, oxfendazole 2.Ivermectin, ivermectin-albendazole combination, tribendimidine, pyrantel pamoate (montherapy or in combination with albendazole, mebendazole, oxantel), nitazoxanide
Control		
Dengue and chikungunya [none]	Anti-viral drugs	1.JNJ‑64281802 2.Chloroquine, ivermectin, balapiravir, ribavirin, celgosivir, UV-4B [23], ivermectin, doxycycline
Buruli ulcer [rifampicin, clarithromycin, moxifloxacin]	New treatment options with reduced treatment duration and lower toxicity, especially for children	1.None 2.Streptomycin, amoxicillin/ clavulanate
Mycetoma [antibiotics for actinomycetoma, antifungals for eumycetoma]	Better treatment regimens (shorter duration, higher efficacy)	1.Fosravuconazole 2.None
Chromoblastomycosis and other deep mycoses [itraconazole, amphotericin B]	Prospectively obtained effectiveness of itraconazole and other antifungals; Improved treatment regimens (shorter duration and increased efficacy)	1.None 2.Fluconazole, isavuconazonium
*Cutaneous leishmaniasis* [pentavalent antimoniate (with or without allopurinol), liposomal amphotericin B, amphotericin B deoxycholate, paromomycin, miltefosine, pentamidine, fluconazole, ketoconazole]	Oral/topical treatment suitable for health center and community level use	1.Potentially LX408 2.Clarithromycin, dapsone (for others see [24–27])
Echinococcosis [albendazole, mebendazole]	Identification of optimal albendazole treatment courses (indicates that drugs with improved efficacy would add value)	1.Oxfendazole, oxantel pamoate 2.None
Foodborne trematodiasis [praziquantel, triclabendazole]	–	1. Oxantel pamoate 2.Tribendimidine, albendazole, mebendazole [28], nitazoxanide
Taeniasis and cysticercosis [albendazole, praziquantel, niclosamide]	Efficacy of current treatment strategies	1.Oxfendazole 2.None
Scabies [ivermectin, topical: permethrin, benzyl benzoate, malathion, sulphur]	Determine efficacy of single dose IVM for programmatic use and safe dose in children < 15 kg, < 90 cm or < 5 years Identify alternative strategies for ivermectin MDA including for loiasis co-endemic areas Evaluate moxidectin	1.Moxidectin 2.None

The literature review and review of the studies in the ICTRP showed that for some NTDs plant extracts/traditional medicines which do not meet our ‘inclusion criteria’ were evaluated. Given the contribution of traditional medicines to drugs in use today (e.g. the artemisinins for malaria [17]) and WHO initiatives to harness such knowledge [18, 19], following the literature for results of these studies will be interesting. For some plant extracts/traditional medicines being evaluated, we included information in Additional file 1.

As for our previous review of drugs in the pipeline for regulatory approval [2], we wanted to ensure to the best of our ability that readers in LMIC can access all references. Therefore, all references are either openly accessible (open access or accessible as author manuscripts e.g. via the US National Institutes of Health’s National Library of Medicine platform (Pubmed Central, PMC), the European Molecular Biology Laboratory European Bioinformatics Institute platform (Europe PMC) or institutional websites, or publications in the WHO Institutional Repository for Information Sharing (WHO IRIS)) or were accessible via HINARI to the WHO staff co-author (ACK). HINARI is a WHO initiative through which not-for-profit institutions in LMIC can obtain free or low cost online access to major journals in the biomedical and related social sciences [20]. Publishers do not provide the same access to the publications in HINARI to all eligible institutions. WHO has a relatively limited level of access and we used WHO staff (ACK) access as the criterion for ‘accessible via HINARI’. Our objective to minimize differences in access to references between readers from rich institutions in high income countries and all other readers has resulted in some relevant literature not being referenced. Hopefully, in the context of increasing attention to Open Access, all journals will, at a minimum, make all content more than 2 years old accessible to all HINARI eligible institutions.

## NTDs targeted for eradication

In the Roadmap, WHO defines eradication as: ‘Permanent reduction to zero of the worldwide incidence of infection caused by a specific pathogen, as a result of deliberate efforts, with no risk of reintroduction’. Documentation of eradication is termed certification [1].

### Yaws

The current strategy is based on PC with azithromycin. For case management, azithromycin is the preferred treatment and benzathine benzylpenicillin the treatment for patients who are suspected to have failed azithromycin treatment and those who cannot be treated with azithromycin [1, 2].

No drugs not already part of current strategies have been evaluated for efficacy against yaws in the past 10 years, and no trials are ongoing or planned as of the records in the ICTRP on 8 February 2023.

### Guinea worm

The current strategy for guinea worm eradication is based on safe drinking water, sanitation and hygiene (WASH) [1, 2].

No drugs have been clinically evaluated for guinea worm in the past 10 years and no trials are planned as per the records in the ICTRP on 8 February 2023.

## NTDs targeted for elimination (interruption of transmission)

In the Roadmap, WHO defines ‘Elimination (interruption of transmission)’ as ‘Reduction to zero of the incidence of infection caused by a specific pathogen in a defined geographical area, with minimal risk of reintroduction, as a result of deliberate efforts; continued action to prevent re-establishment of transmission may be required’. Documentation of elimination of transmission is termed verification [1].

### Leprosy

Drugs currently recommended for case management include rifampicin, dapsone, clofazimine, clarithromycin, minocycline or a quinolone (ofloxacin, levofloxacin or moxifloxacin). Rifampicin is recommended for chemoprophylaxis [1, 2].

A recent systematic review identified no regimens better than the WHO recommended regimens. With the exception of regimens including sparfloxacin or acedapsone, all regimens evaluated were based on drugs already recommended by WHO [21]. Our ICTRP search on 8 February 2023 did not identify studies with other drugs for potential off-label use.

### Human African trypanosomiasis due to *Trypanosoma brucei gambiense*

The drugs currently recommended for case management include fexinidazole, eflornithine-nifurtimox combination and pentamidine [1, 2].

No trials with other drugs or not conducted in view of regulatory registration [2] have been conducted in the past 10 years, are currently ongoing or planned as per the records in the ICTRP on 8 February 2023.

### Onchocerciasis

The current strategy for elimination of transmission of *Onchocerca volvulus* is based on PC with ivermectin [1, 2].

#### Ivermectin-albendazole and ivermectin-diethylcarbamazine-albendazole combination

Addition of albendazole to bi-annual or annual single-dose ivermectin treatment did not significantly affect the proportion of adult female worms with normal embryogenesis, dead macrofilariae or of individuals with detectable skin microfilariae levels (ISRCTN50035143, Clinicaltrials.gov identifier NCT03238131) [29].

The superiority of ivermectin-diethylcarbamazine-albendazole over diethylcarbamazine-albendazole for LF resulted in a conditional WHO recommendation for use of this combination for LF elimination in specified situations and where neither onchocerciasis nor loiasis is endemic [4]. A pilot-study evaluating ivermectin-diethylcarbamazine-albendazole treatment of *O. volvulus*-infected individuals completed collection of the data for the primary outcome (rates of severe adverse events (AEs) and percentage of worms killed/sterilized) in January 2022. Macrofilariae evaluation is ongoing (NCT04188301, Pan African Clinical Trials Registry identifier (PACTRI) PACTR201906665550709) [30]. Eligible individuals were treated 6 months earlier with ivermectin to reduce microfilariae levels and thus the probability of adverse reactions to diethylcarbamazine [31]. The severity of such reactions is a function of pre-treatment microfilaria levels and diethylcarbamazine dose [32–34].

#### Antibiotics targeting the *O. volvulus* Wolbachia symbiont

Research into the role of endosymbiotic *Wolbachia* bacteria in development of filarial parasites (*O. volvulus*, *Wuchereria bancrofti*, *Brugia malayi*, *B. timori*, *Mansonella perstans*) and the effect of antibiotics on their fertility and viability dates back to the 1990s [35]. Clinical proof of concept for the utility of targeting *Wolbachia* for treatment of filarial infections was obtained with doxycycline for *O. volvulus*: *Wolbachia* depletion 6 months after 4 or 6 weeks of treatment with 200 mg or 100 mg doxycycline/day was a predictor of female *O. volvulus* worm death or sterility [36–41].

The long doxycycline treatment duration required is a challenge for large-scale use outside clinical or pilot implementation studies. Furthermore, neither onchocerciasis nor LF meets current doxycycline labelling criteria for use in children < 9 years, pregnant or breastfeeding women which have been questioned for young children and pregnant women [42]. These factors continue to drive evaluation of other antibiotics already approved for human use including minocycline [43, 44] and rifapentine-moxifloxacin combination treatment [45] as well as discovery of new antibiotics and preclinical and clinical development of emerging drug candidates. This research has recently been reviewed [2, 22].

Rifampicin and rifapentine are rifamycins and ansamycins developed in the 1960s. They are active against gram-positive and, to a lesser extent, gram-negative bacteria and are an important element of multi-drug tuberculosis treatment. Rifamycins block the early stages of DNA-dependent RNA synthesis [46].

Rifapentine-moxifloxacin combination: Based on animal model results [47], an open label, proof-of-concept phase 2 trial evaluated the *Wolbachia* depleting effect of 7 or 14 days of moxifloxacin 400 mg/day + rifapentine 900 mg/day in Ghana (ISRCTN43697583).

Rifampicin: In a proof-of-concept study, 10 mg/kg/day rifampicin for 2 (*n* = 12) or 4 (*n* = 16) weeks resulted in 0% (0/23) and 18% (2/11) live macrofilariae without *Wolbachia* 18 months after treatment compared to 1% (1/88) in the concurrent untreated control and 82% (9/11) in a historical 6-week 100 mg/day doxycycline control [48]. Considering the dose-response relationship in animal models [49] and the tolerability of rifampicin up to 35 mg/kg [50], two trials evaluating the efficacy of rifampicin in combination with albendazole for both onchocerciasis and lymphatic filariasis were initiated (Table 1).

#### Drugs for safe reduction of *Loa loa* microfilaraemia in onchocerciasis and LF co-endemic areas

Ivermectin mass drug administration is currently the main strategy for control and elimination of onchocerciasis and ivermectin is also a component of the strategy for control and elimination of LF. Since ivermectin can result in severe and potentially deadly adverse reactions in individuals with high *Loa loa* microfilaraemia [51], loiasis co-endemicity is a significant obstacle to elimination of *O. volvulus* transmission and elimination of LF as a public health problem in loiasis co-endemic areas in Africa [52]. Alternative strategies are required in *L. loa* co-endemic areas [53, 54]. These could include treatments which target Wolbachia (*L. loa* does not harbour Wolbachia symbionts [55]) as well as treatment with drugs which reduce *L. loa* microfilaraemia safely before ivermectin treatment.

Imatinib is a protein-tyrosine kinase inhibitor registered in the US since 2001 for treatment of leukaemia [56]. Non-clinical studies identified imatinib as a micro- and macrofilaricidal candidate drug [57, 58]. A case report suggested safe *L. loa* microfilaraemia reduction [59]. A pilot study comparing the effect of a single dose of 200 mg, 400 mg, 600 mg or placebo on *L. loa* microfilaraemia was terminated based on a planned interim analysis demonstrating futility of the intervention (NCT02644525). Protein kinase inhibitors are in non-clinical evaluation for *Fasciola hepatica* [60] and schistosomiasis [61].

Levamisole is an imidazothiazole and a nicotinic acetylcholine receptor (nAchR) receptor agonist [62] with a mechanism of action not fully understood and a long history of use for intestinal helminth control [63, 64]. It is on the WHO Essential Medicines List (EML) for ascariasis. The safe but only transient *L. loa* microfilaraemia reduction after a single 2.5 mg/kg levamisole dose should motivate studies of higher doses and longer treatment [65]. Table 1 shows studies completed but not yet published.

## NTDs targeted for elimination as a public health problem

In the Roadmap WHO defines ‘Elimination as a public health problem’ as ‘Reduction to zero of the incidence of infection caused by a specific pathogen in a defined geographical area, with minimal risk of reintroduction, as a result of deliberate efforts; continued action to prevent re-establishment of transmission may be required’. Documentation of elimination as a public health problem is termed validation [1].

### Rabies

Anti-infectives are not part of the current strategy [1, 2]. No anti-infectives have been trialed or studies registered in the past 10 years.

### Trachoma

The current core strategic intervention is PC with azithromycin [1, 2]. No other drugs have been clinically evaluated in the past 10 years, and no trials are currently ongoing or planned as per the records in the ICTRP on 8 February 2023.

### Chagas disease

The drugs currently recommended for case management are benznidazole and nifurtimox [1, 2].

The history and current pipeline for Chagas disease treatment have been recently reviewed [66–68]. The current pipeline for off-label use includes azoles (itraconazole, fluconazole, ketoconazole, posaconazole and ravuconazole) and allopurinol, primarily as combination treatment with nifurtimox or benznidazole. A detailed description of these and other compounds that have been or are being evaluated and the rationale for their evaluation for Chagas disease has been provided recently [66].

Disulfiram (tetraethylthiuram disulphide) is a carbamoyl derivative, approved for alcohol aversion therapy: it inhibits acetaldehyde dehydrogenase resulting in accumulation of the ethanol metabolite acetaldehyde, which causes a range of unpleasant symptoms (Disulfiram | C10H20N2S4—PubChem (nih.gov), accessed 12 March 2022). Disulfiram inhibits 20S proteasome activity [69] and is a candidate for repurposing as a cancer drug [70], antibiotic [71, 72] and anti-leishmanial drug [73]. Its antitrypanosomal activity was reported in 1996 [74]. The ongoing study in Brazil is evaluating its safety and anti-trypanosomal efficacy in combination with benznidazole in chronic Chagas disease (Table 1).

### Human African trypanosomiasis caused by *Trypanosoma brucei rhodesiense*

The drugs currently recommended for case management are suramin for first-stage and melarsoprol for second-stage *Trypanosoma brucei rhodesiense* human African trypanosomiasis (HAT) [1, 2].

No trials with other drugs or not conducted in view of regulatory registration [2] have been conducted in the past 10 years, are currently ongoing or planned as per the records in the ICTRP on 8 February 2023.

### Visceral leishmaniasis

Drugs recommended for management of cases of visceral leishmaniasis include pentavalent antimonials, liposomal amphotericin B, paromomycin and miltefosine [1, 2].

No studies evaluating the efficacy and safety of drugs not already included in current strategies and not conducted in view of regulatory registration [2] for the treatment of visceral leishmaniasis have been published or conducted over the past 10 years, are currently being conducted or planned as per the records in the ICTRP on 8 February 2023.

### Lymphatic filariasis

The current strategy for control of LF as a public health problem is PC with albendazole alone or in combination with ivermectin and/or diethylcarbamazine depending on co-endemicity of onchocerciasis and/or loiasis and PC history [1, 2].

*Wuchereria bancrofti*, *B. malayi* and *B. timori* harbour Wolbachia. The status of research into antibiotics to treat filarial diseases has been recently reviewed [22]. Two studies evaluating antibiotics are currently ongoing or in preparation (Table 1).

### Schistosomiasis

Both strategies recommended by WHO to eliminate schistosomiasis as a public health problem (PC and ‘test and treat approaches’, depending on endemicity and PC history) use praziquantel [1, 2].

#### Artemisinins (artemisinin derivatives)

Research into the effect of artemisinins on schistosomes was initiated in China in the 1980s and identified activity against juvenile stages of *Schistosoma japonicum* [75–78].

Evaluation of the effect of treatment of malaria patients on co-infection with schistosomiasis suggested that treatment with artemisinin-based combination therapy for malaria provides benefits for infection with *Schistosoma mansoni* [79] and *S. haematobium* [80]. Systematic reviews and meta-analyses concluded that the combination of artemisinins with praziquantel, but not artemisinins alone, can lead to higher cure rates (CR) than praziquantel alone but that studies with less risk of bias are needed for definitive conclusions [81–84]. A higher effect of artemisinins and praziquantel combination treatment may be due to the fact that artemisinins have activity against juvenile stages while praziquantel affects adult worms with little activity against the juvenile stages [76, 85–87].

Randomized studies conducted since these reviews include a single-blinded (participant and laboratory staff) exploratory study in Côte d’Ivoire (ISRCTN 63657086) [87] and an open-label non-inferiority study in Tanzania (PACTR201612001914353) [87]. In Côte d’Ivoire, 12–17-year-old adolescents infected with *S. haematobium* or *S. mansoni* were randomized to three daily doses of Synriam (150 mg arterolane plus 750 mg piperaquine), a single dose of praziquantel 40 mg/kg plus three daily doses of Synriam or a single dose of 40 mg/kg praziquantel (or to a single dose of moxidectin). The number of children included in the analysis ranged from 26 to 30. CRs (95% confidence interval) 3 weeks after the last dose of the combination vs. praziquantel treatment for *S. haematobium* were 60.0% (40–80%) vs. 38.5% (20–60%) and for *S. mansoni* 27.0% (10–50%) vs. 27.6% (10–50%). Egg reduction rates (ERR), considered a more suitable indicator for drugs for PC for schistosomiasis and STH [88], calculated based on the geometric mean egg counts, were 96% (80–100%) vs. 93.5% (80–100%) for *S. haematobium* and 77.6% (50–110%) vs. 87.5% (80–100%) for *S. mansoni* after combination vs. praziquantel treatment. Treatment with Synriam alone or with moxidectin was not efficacious. Further studies are needed before definitive conclusions can be drawn, including follow-up on the CR in the praziquantel monotreatment arms [87]. In Tanzania, *S. mansoni*-infected 7–17-year-old children were randomized to a single dose of 40 mg/kg praziquantel plus 4 mg/kg dihydroartemisinin per day and 18 mg/kg piperaquine per day for 3 days (as per WHO guidelines for treatment of uncomplicated malaria [89]) or a single dose of 40 mg/kg praziquantel (PACTR201612001914353). CRs (95% confidence interval) in the combination (*n* = 298) vs. praziquantel treatment group (*n* = 341) were 88.3% (84.1–91.4%) vs. 81.2% (76.7–85.0%), respectively, 3 weeks after treatment and 81.9% (77.1–85.8%) vs. 63.9% (58.7–68.8%), respectively, 8 weeks after treatment. ERRs, calculated based on arithmetic mean egg counts, also showed a smaller treatment difference between the combination vs. praziquantel treatment groups 3 weeks after treatment [95.3% (92.9–97.7%) vs. 95.0% (92.7–97.3%)] than 8 weeks after treatment [93.6% (90.8–96.4%) vs. 87.9% (84.4–91.4%)] [90].

The effect of treatment of *S. haematobium* co-infected 7–18-year-old patients for uncomplicated malaria on egg excretion and cure rates was evaluated in Gabon (NCT04264130). Median (interquartile range) egg excretion rates 4 and 6 weeks after treatment, respectively, were 100% (17–100%) and 65% (− 133–100%) after artesunate-pyrinaridine treatment (*n* = 21) and 35% (− 250–70%) and 65% (− 65–79%) after artemether-lumefantrine treatment (*n* = 18). Cure was only observed after artesunate-pyronaridine treatment (56% and 37% at 4 and 6 weeks after treatment, respectively). [91].

A trial evaluating the effect of artesunate-mefloquine combination on schistosomiasis [92] has completed recruitment in Senegal (NCT03893097) but results have not yet been published.

Any decisions to include artemisinins or other anti-malarial drugs in strategies for elimination of schistosomiasis as a public health problem will have to consider the risk to malaria programmes.

### Soil-transmitted helminthiasis (STH) including strongyloidiasis

The WHO recommended strategy to eliminate STH and strongyloidiasis as a public health problem is based on PC with albendazole or mebendazole targeting children from 12 months through school age, non-pregnant adolescent girls and women of reproductive age, pregnant women in second and third trimester and lactating women [1, 2]. Table 1 shows the currently ongoing study.

#### Ivermectin and ivermectin combinations

WHO is preparing new guidelines for PC for STH and strongyloidiasis. Systematic review of all available evidence, a standard element of guideline preparation [93], is currently expected to result in addition of ivermectin to PC with albendazole or mebendazole in areas where prevalence of *Strongyloides stercoralis* exceeds 10% and in areas with high prevalence of *Trichuris trichiura* infection [1]. The results of the systematic review will be appended to the guideline.

Strongyloidiasis is one of the two indications for which oral ivermectin has regulatory approval (single dose of 200 µg/kg), but only for children weighing at least 15 kg. This is also the lower weight limit for the other US FDA-approved indication, onchocerciasis (single dose of 150 µg/kg) [94]. Minimum weight and height for which ivermectin is approved for onchocerciasis and LF by the French Agence nationale de sécurité du médicament et des produits de santé are 15 kg and 90 cm [9].

This weight or height limit drives exclusion of smaller children from PC including ivermectin as well as from ivermectin treatment in most trials evaluating ivermectin for its efficacy against STH and strongyloidiasis (and e.g. scabies) either completely (e.g. ongoing studies NCT03605758, TCTR20190111001) or inclusion with treatments other than ivermectin (e.g. albendazole for STH and strongyloidiasis [95], permethrin for scabies [96]). Given the burden of disease, an important consideration during WHO guideline development will be the lower age limit (based on age, height, or weight) of eligibility for ivermectin treatment and the relevant dose. Identification of a safe and efficacious dose for smaller children is also important for use of ivermectin for other infections, notably scabies [97], and may impact eligibility criteria for trials of new drugs in which ivermectin is a comparator.

Reviews to date of safety data for children < 5 years or weighing < 15 kg treated with ivermectin for a variety of infections with doses around 200 µg/kg identified no safety concerns [98, 99]. A dose-finding PK, safety and efficacy study (ISRCTN15871729) in Côte d’Ivoire in *T. trichiura*-infected children 2–5 years (*n* = 80, 100 or 200 µg/kg) and 6–12 years (*n* = 120, 200, 400 or 600 µg/kg) old and adults (*n* = 11, 200 µg/kg) showed lower dose-dependent dose-adjusted exposures in children than adults. AE data collected to 72 h and indicators of hepatic and renal function 72 h after treatment identified no safety concerns [100, 101]. A randomized controlled trial in Honduras investigated the PK, efficacy and safety of high-dose ivermectin in *T. trichiuria*-infected children 2–14 years old weighing at least 15 kg. Fifty-six and 58 children, respectively, received 600 µg/kg ivermectin with 400 mg albendazole once or for 3 days. No safety concerns and no differences in IVM blood concentrations between children with and without AEs were identified [102]. Two population PK modelling studies of ivermectin concentration data obtained in *T. trichiura* [101] and scabies-infected children [103] proposed a 3 mg dose of ivermectin for children 75–90 cm [104] or children 2–4 years old weighing 10–15 kg [103]. The currently available formulation is a 3 mg tablet. Two studies evaluating ivermectin PK and safety in smaller children with scabies infection are planned (NCT04332068, NCT05500326). A trial evaluating the pharmacokinetics and pharmacodynamics of three daily doses of ivermectin in paediatric dengue patients (400 µg/kg/day or 600 µg/kg/day, minimum participant weight 15 kg) has been completed (NCT03432442) but has not yet been published (Table 1).

In the ivermectin paediatric dose-finding PK, safety and efficacy study (ISRCTN15871729), *T. trichiura* CR and ERR 14–21 days after treatment were not different after up to 400 µg/kg ivermectin compared with after placebo and were low or moderate even after 600 µg/kg. CR and ERR for co-incidental *Ascaris lumbricoides* (between 8 and 14 participants) were around 90–100% and 100%, respectively [101].

Better results were obtained when ivermectin was added to albendazole treatment. In a double-blind randomized trial in Côte d’Ivoire, Laos and Pemba Island, Tanzania, 6–60-year-old individuals with at least 100 *T. trichiura* eggs/g stool were treated with a single dose of 200 µg/kg ivermectin plus 400 mg albendazole or 400 mg albendazole. CRs after ivermectin + albendazole were significantly higher than after albendazole alone in Laos (66% vs. 8%) and on Pemba Island (49% vs. 6%) but similar in Côte d’Ivoire (14% vs. 10%). The same was true for the effect of the combination vs. monotherapy on geometric mean-based ERRs, which were 99% vs. 69% in Laos, 98% vs. 57% in Pemba Island and 70% vs. 64% in Côte d’Ivoire. The reason for the difference in efficacy observed in Laos and on Pemba Island vs. Côte d’Ivoire remains to be investigated. No safety concerns were identified [105]. In the study in Honduras, the efficacy data obtained from 117 children showed *T. trichiura* CR of 88.6% and 100% and ERR of 96.7% and 100%, respectively, 14–21 days after treatment with 600 µg/kg ivermectin plus 400 mg albendazole once or for 3 consecutive days compared to CR of 4.2% and 33.3% and ERR of 47.7% and 72.1% after a single or three daily doses of 400 mg albendazole, respectively. The AE profile in all four treatment arms indicated acceptable tolerability [102].

#### Tribendimidine

Tribendimidine is a symmetrical diamidine and, like levamisole, oxantel and pyrantel pamoate, a nAchR agonist [106]. The mechanism of action may depend on the nematode, trematode or cestode species [107, 108].

Tribendimidine is a broad-spectrum anthelmintic and was registered for STH treatment in China in 2004 [107, 109, 110]. This motivated work towards registration elsewhere, including in the USA [111], spearheaded by the collaboration between the Swiss Tropical and Public Health Institute and the Chinese Center for Diseases Control and Prevention. Non-clinical pharmacology and clinical studies against a wide range of helminths were reviewed in 2013 [107]. Additional file 1: Table S2 provides the CRs and ERRs for *T. trichuris*, hookworm and *A. lumbricoides* reported for tribendimidine mono- and combination treatment since 2013 together with the results for other treatment arms included in the studies [111–114]. Tribendimidine should be further evaluated as a useful addition to the limited number of drugs available for PC, in particular for *S. stercoralis* [115] and in combination treatment against hookworm [116, 117]. None of the studies identified AEs precluding further evaluation.

#### Pyrantel pamoate and pyrantel pamoate combinations

Pyrantel is a tetryhydropyrimidine, introduced to the small animal market for the control of hookworm and roundworm in the 1970s [118]. Like oxantel, tribendimidine and levamisole, pyrantel is a nAChR agonist. Pyrantel and levamisole activate the L-subtype [62]. Pyrantel pamoate (also known as embonate, see [2]) is on the 2021 WHO EML for ascariasis, trichuriasis, enterobiasis and hookworm and on the EML for children for intestinal helminths [119].

The few placebo- or active-controlled studies available suggest that the efficacy of a single 10 mg/kg dose pyrantel pamoate dose is comparable to that of single albendazole or mebendazole doses for *A. lumbricoides* but unsatisfactory for hookworm and very low for *T. trichiura* [111, 120], consistent with veterinary experience [118]. Combination with albendazole did not increase CR or ERR [121]. Combination of pyrantel pamoate with albendazole and oxantel pamoate resulted in CR and ERR for *A. lumbricoides*, *T. trichiura* and hookworm superior to those of albendazole-oxantel, pyrantel-oxantel and mebendazole-pyrantel-oxantel combinations [122].

#### Nitazoxanide

Nitazoxanide is a nitrothiazole benzamide first described in 1975 and available in some Latin American countries since 1996 [123]. Nitazoxanide was approved by the US FDA in 2002 for use in children 1–11 years old with diarrhoea due to *Cryptosporidium parvum* and *Giardia lamblia* [124, 125]. Nitazoxanide is a pro-drug with high oral bioavailability that is metabolized to the active drug tizoxanide, which is active against a broad spectrum of intestinal protozoa, helminths and anaerobic bacteria. Studies in *Caenorhabditis elegans* showed that nitazoxanide affects a subunit of the glutamate-gated chloride channels through which ivermectin acts, that other mechanisms are likely involved and that nitazoxanide acts synergistically with albendazole and pyrantel Somvanshi et al. [126]. Thiazolides, including nitazoxanide, have activity against many DNA and RNA viruses [127–129]. Nitazoxanide has been evaluated in vitro against mycobacteria [130–132] and is being investigated as a cancer drug (as are other antiparasitic drugs [133].

Pharmacology and early studies of efficacy in approved indications (cryptosporidiosis, giardiasis) and off-label uses (including *A. lumbricoides*, *T. trichiura*, *S. stercoralis*, *F. hepatica*, *Taenia saginata, Enterobius vermicularis, Hymenolepis nana, Ancyclostoma duodenale*) have been reviewed [123, 134]. In a double-blind, randomized trial (ISRCTN83836427) in 533 Tanzanian children, 1000 mg nitazoxanide alone or in combination with 400 mg albendazole did not increase *T. trichiura* or hookworm CR and ERR beyond that achieved with 400 mg albendazole alone. Children receiving nitazoxanide had significantly more adverse events than children receiving placebo, but most adverse events were transient and mild [135].

## NTDs targeted for control

WHO defines ‘Control’ as ‘Reduction of disease incidence, prevalence, morbidity and/or mortality to a locally acceptable level as a result of deliberate efforts; continued interventions are required to maintain the reduction. Control may or may not be related to global targets set by WHO [1].

### Dengue and chikungunya

In the absence of antiviral drugs, WHO recommends symptomatic treatment for case management and vaccination for people with previous laboratory-confirmed dengue infection [1, 2].

A review of drugs in clinical development for dengue, identified through search of the clinical trials in the ICTRP as of 2015 (including the anti-parasitic drugs chloroquine and ivermectin, anti-inflammatory agents corticosteroids and statins, anti-viral drugs balapiravir and ribavirin, iminosugars celgosivir and UV-4B, and traditional medicines *Cissampleos pareira* extracts and *Carica papaya* extracts) concluded that promising treatments are yet to be identified and that significant investment is required to identify safe, effective and inexpensive drugs [23]. An update on anti-dengue drug discovery has been provided recently [136].

The vast majority of clinical trials do not evaluate anti-infective drugs registered for human use for an indication other than the NTD for which they are trialed and thus do not meet our inclusion criteria. Given the extent of research with traditional medicines which does not meet our inclusion criteria (*Cocculus hirsutus* extracts [137, 138], *C. papaya* extracts [139–141], Ganghuo Kanggan decoction (GHKGD) [142], *Eupatorium perfoliatum* [143, 144]) we provided short summaries in Additional file 1.

Ivermectin has been shown to inhibit the replication of all four DENV serotypes in vitro [145]. The rationale for its evaluation for dengue has been provided previously [23]. A randomized, placebo-controlled trial of three daily dose of 400 µg/kg of ivermectin in adult dengue patients showed that the incidence of adverse events was comparable in the placebo and ivermectin treatment group. There was no difference in viraemia clearance time but ivermectin accelerated plasma nonstructural protein1 clearance [146]. A trial evaluating the pharmacokinetics and pharmacodynamics of three daily doses of ivermectin in paediatric dengue patients (400 µg/kg/day or 600 µg/kg/day, minimum participant weight 15 kg) has been completed (NCT03432442) but has not yet been published.

Doxycycline antiviral activity has been shown against flaviviruses [147]. Evaluation of the efficacy of doxycycline treatment for dengue is planned (Table 1, CTRI/2021/09/036661).

### Buruli ulcer

The current core strategic intervention is case management with rifampicin and clarithromycin or moxifloxacin [1, 2].

A randomized controlled trial in Ghana in 297 participants at least 5 years of age with PCR-confirmed *Mycobacterium ulcerans* infection and lesions of < 10 cm diameter did not identify a significant difference in the percentage of participants with healed lesions between 8 weeks of daily treatment with extended-release clarithromycin (15 mg/kg oral) and rifampicin (10 mg/kg oral) and 8 weeks of daily streptomycin (intramuscular 15 mg/kg) and rifampicin (10 mg/kg oral) treatment [148]. Two studies evaluating the effect of adding amoxicillin/clavulanate to the standard regimen are ongoing or planned (Table 1).

### Actinomycetoma and eumycetoma

The current case management strategy for actinomycetoma is long-term treatment with antibiotic combinations and wound cleaning/dressing. The types of antibiotics are not specified in the Roadmap. For eumycetoma, case management with antifungals (mainly itraconazole) and surgery combined with would cleaning/dressing is recommended [1, 2].

Early detection and treatment are critical, but rare given that mycetoma affects primarily poor communities in remote areas in the LMICs of the ‘mycetoma belt’ (latitudes of 15^◦^ South and 30^◦^ North) [149, 150]. Available treatment options are ineffective and have an unfavourable safety profile and required treatment duration is unsuitable for the health system context [151]. Approaches to mycetoma management have been reviewed recently and the need for improved tools emphasized [151–156].

No clinical trials to assess the safety and efficacy of drugs in actinomycetoma have been registered in the past 10 years as per records in the ICTRP as of 8 February 2023.

Beyond the proof-of-concept study comparing fosravuconazole vs. itraconazole for eumycetoma in Sudan (NCT03086226) [2, 151], no other drug trials have been registered in the past 10 years.

The challenges clinical trials for mycetoma face have been outlined recently [157].

### Chromoblastomycosis and other deep mycoses

For management of chromoblastomycosis and other deep mycoses, the Roadmap specifies that there is no gold standard and lists a number of treatment options, including physical therapies, immune adjuvants, and surgery of minor lesions and treatment with the antifungal drug itraconazole [1, 2]. Itraconazole is included in the WHO EML and the WHO EML for children for chromoblastomycosis and paracoccidioidomycosis [119].

In the last 10 years the drugs fluconazole and isavuconazonium sulphate have been evaluated in clinical trials against deep mycosis.

Fluconazole is a synthetic triazole antifungal agent registered in the US for vaginal, oropharyngeal and oesophageal candidasis and for cryptococcal meningitis [158]. The Committee for Medicinal Products for Human Use of the European Medicines agency recommended use in the European Union for mucosal and invasive candidiasis, genital candidiasis (trush), cryptococcal meningitis, dermatomycosis, coccidiodomycosis and onychomycosis [159]. Fluconazole is a highly selective inhibitor of the fungal cytochrome P450-dependent enzyme lanosterol 14-α-demethylase, which converts lanosterol to ergosterol. The subsequent loss of normal sterols results in accumulation of 14-α-methyl sterols in fungi and may be responsible for the fungistatic activity of fluconazole. Mammalian cell demethylation is much less sensitive to fluconazole inhibition [158]. Given that fluconazole is only fungistatic, efforts are underway to identify whether it can be combined with other agents to achieve a fungicidal effect [160, 161]. In 2014, a Pfizer-sponsored observational prospective case-only study (*N* = 27) (NCT01680458) evaluated the safety and efficacy of fluconazole in the treatment (*n* = 2) and prophylaxis (*n* = 25) of deep mycoses in children < 4 weeks to 7 years. The results have been posted on the clinicaltrials.gov website record, but we were unable to identify a peer-reviewed publication as of December 2022.

Isavuconazonium is a prodrug of the active drug isavuconazole, a triazole with a broad spectrum of activity against yeasts, moulds and dimorphic fungi [162]. It has been registered since 2015 in the US and Europe for treatment of invasive aspergillosis and invasive mucormycosis in adults. Like fluconazole, isavuconazole inhibits the cytochrome P450-dependent lanosterol 14-α-demethylase and thus the synthesis of ergosterol, a critical component of the fungal cell membrane [162–164]. Isavuconazole has also been evaluated for other invasive fungal infections including cryptococcosis, paracoccidioses and chronic pulmonary aspergillosis [165, 166].

Given that these studies were sponsored by pharmaceutical companies, it is possible that ultimately regulatory registration for additional indications will be sought.

### Cutaneous leishmaniasis

The cutaneous leishmaniasis (CL) case management strategy depends on factors including the disease, concomitant pathologies, parasite species, location and national guidelines. Topical/intralesional treatment approaches include pentavalent antimonials, paromomycin/methylbenzethonium chloride, cryotherapy and thermotherapy. Drugs for systemic treatment include fluconazole, ketoconazole, liposomal amphotericin B, amphotericin B deoxycholate, pentamidine, pentavalent antimonials (with or without allopurinol), paromomycin and miltefosine [1, 2].

Most studies evaluating new treatment options identified in the ICTRP are outside the scope of our manuscript since they evaluate the effect of intralesional and topical treatments. Treatment for CL overall and CL in the Americas has been reviewed recently including experimental treatments [24–27]. Oral treatments with recent publications not covered in these reviews are included here.

Around 1990, oral dapsone (2 mg/kg for 6 weeks) was shown to have efficacy in the treatment of CL in India [167–169]. Oral dapsone is used in Indian hospitals (see e.g. [170]). Evaluation of a case series (*N* = 11) in Colombia resulted in the conclusion that dapsone efficacy was not satisfactory against *Leishmania (V) panamensis* [171]. A study comparing the effect of intralesional injection of glucantime without and with oral dapsone in Iran has been completed, but we were unable to identify a publication (IRCT20140211016554N4). In Pakistan, a randomized trial compared intramuscular meglumine antimoniate (15 mg/kg/day, total ≤ 15 ml) to healing or for 40 days and oral dapsone (2.5 mg /kg/body weight/day, ≤ 200 mg/day) to healing or for 80 days in patients with biopsy-confirmed CL. Post-treatment blinded efficacy evaluation showed 51–75% reduction and 76–100% reduction in lesion size in 21/50 and 21/50 patients, respectively, after meglumine antimoniate and in 7/50 and 33/50 patients, respectively, after oral dapsone treatment [172].

Clarithromycin is a semi-synthetic macrolide with in vitro activity shown against *Leishmania major* [173]. In a pilot study in Iran, previously untreated patients were randomly assigned to clarithromycin (*N* = 10, 500 mg twice a day for 2 months, mean ± standard deviation of lesion number 5 ± 4.3 and induration size 15.47 ± 15.6 mm) or three injections of glucantime (*N* = 10, 20 mg/kg/day for 20 days; *N* = 10, lesion number 3 ± 2.8, induration size 19.81 ± 13 mm). Three, 6 and 12 months after treatment, lesions had disappeared in all patients in the clarithromycin group and the mean number of lesions in the glucantime group was 0.17 ± 0.04 with a mean induration size of 1.59 ± 6.8 mm [174].

Given the number of studies in the ICTRP evaluating traditional medicines, information on plant extracts with anti-leishmanial activity or activity against other infectious agents demonstrated in in vitro or in vivo studies is included in Additional file 1.

### Echinococcosis

Albendazole and mebendazole are the drugs currently recommended for case management of echinococcosis [1, 2].

No studies of other drugs have been published or conducted over the past 10 years, are ongoing or planned as per the records in the ICTRP on 8 February 2023.

### Foodborne trematodiasis

The current strategies for PC and case management are based on praziquantel and triclabendazole [1, 2].

### Clonorchiasis and opistorchiasis (small liver flukes)

Comparative trials evaluating the effect of tribendimidine, albendazole, mebendazole and praziquantel alone or in combination on *Clonorchis sinensis*, *Opisthorchis viverrini* and *O. felineus* have recently been reviewed. The review found that praziquantel is highly efficacious, recommended further evaluation of tribendimidine as a potential alternative treatment option and concluded that too few high quality studies are available to support definitive conclusions about the benefit of 5–7 days of treatment with albendazole [28].

#### Fascioliasis

Nitazoxanide at a dose of 500 mg orally every 12 h for 7 days was partially effective in 30% of patients with clinical manifestations of acute fascioliasis who had failed triclabendazole treatment suggesting that further clinical evaluation of nitazoxanide for fascioliasis should be conducted [175].

#### Paragonimiasis

No trials focussed on paragonimiasis were identified.

### Taeniasis and cysticercosis

The current strategies for PC and case management are based on praziquantel, niclosamide and albendazole [1, 2]. No studies of other drugs have been published or conducted over the past 10 years, are ongoing or planned as per the records in the ICTRP on 8 February 2023.

### Scabies

Core elements of current strategies for control of scabies include PC and case management with ivermectin and with topical scabicides for children too small to be eligible for ivermectin [1, 2]. Identification of an efficacious and safe dose of ivermectin for small children is a priority, as are evaluation of moxidectin and identification of an alternative treatment strategy for loiasis co-endemic areas [1, 2]. The current status of research for a safe ivermectin dose for children has been provided in the context of clinical studies drugs against STH and strongyloidiasis.

## Conclusions

Considering both the small anti-infective drugs in at least Phase 2 clinical development for regulatory approval [2] and the small anti-infective drugs/drug combinations being evaluated for repurposing, the pipeline for drugs and drug combinations for NTDs is, unsurprisingly but still disappointingly, small (Table 2).

Given the factors that impact the feasibility of advances in discovery of new drugs, their pre-clinical characterization to qualify them for clinical development and funding and other challenges clinical trials for NTDs face, whether with new molecular entities or drugs that could be repurposed, not only increased advocacy for research into new treatment options but also additional paths to new treatment options are needed.

One promising path is ‘CURE ID’, initiated by the US FDA in collaboration with the US National Institutes of Health’s National Center for Advancing Translational Sciences. WHO joined the collaboration [176] as did the Infectious Diseases Society of America, the Critical Path Institute and the Drugs for Neglected Diseases initiative. CURE-ID is a web-based platform, accessible on the computer or via a mobile app, in which healthcare providers can share their experience with repurposing drugs for diseases that lack effective and safe treatments via succinct easy to fill deidentified case report forms. In addition, users can view and participate in discussions and stay up to date through a newsfeed that is updated daily providing information on case reports submitted, discussion posts, clinical trials, the latest infectious disease journal articles and events. The platform is searchable by disease/infectious agent. Analysis of the pooled experience reported may identify treatments that can advance into clinical trials. Additional file 1: Table S4 includes links to further information.

### Supplementary Information

**Additional file 1: Table S1.** Source registries and last data file import into the WHO Clinical Trials Registry Platform as of 8 February 2023. **Table S2.** Cure and egg reduction rates in studies of the efficacy of tribendimidine alone and in combination and in comparator treatment arms against intestinal helminths. Background information on traditional medicines evaluated for Dengue, Background information on traditional medicines evaluated for cutaneous leishmaniasis, **Table S3.** Resources on CURE ID.

## Data Availability

Not applicable.

## Abbreviations

ADME: Absorption, distribution, metabolism, elimination
APOC: African Programme for Onchocerciasis Research
CR: Cure rate
CTRI: Clinical Trials Registry India
DNDi: Drugs for Neglected Diseases initiative
EDCTP: European & Developing Countries Clinical Trials Partnership
EMA: European Medicines Agency
EML: WHO Essential Medicines list
ERR: Egg reduction rate
HAT: Human African trypanosomiasis
ICTRP: WHO International Clinical Trials Registry Platform
ISRCTN: Clinical study registry managed by Biomed Central, Part of Springer Nature
LMIC: Low- and middle-income countries
LF: Lymphatic filariasis
MDGH: Medicines Development for Global Health
NCT: Prefix of clinical trial numbers in the data base of privately and publicly funded clinical studies conducted around the world by the US National Institute of Health
NDA: New Drug Application
NTD: Neglected Tropical Disease
PACTRI: Pan African Clinical Trials Registry identifier
PC: Preventive chemotherapy
PK: Pharmacokinetics
RBR: Registro Brasileiro de Ensaios Clinicos
R&D: Research and development
SRA: Stringent regulatory authority (for definition see https://extranet.who.int/pqweb/content/glossary)
STH: Soil transmitted helminths/helminthiasis
STPH: Swiss Tropical and Public Health Institute
TDR: UNICEF/UNDP/World Bank/WHO Special Programme for Research and Training in Tropical Diseases
US FDA: United States of America Food and Drug Administration
WASH: Safe drinking water, sanitation and hygiene
WHO: World Health Organization
