# Supplementary material for: The pipeline for drugs for control and elimination of neglected tropical diseases: 2. Oral anti-infective drugs and drug combinations for off-label use
Source: Parasit Vectors. 2023 Oct 31;16:394. doi: 10.1186/s13071-023-05909-8 (PMC10619278; doi:10.1186/s13071-023-05909-8)
Supplement: Supplementary file 1 — Additional file 1: Table S1. Source registries and last data file import into the WHO Clinical Trials Registry Platform as of 8 February 2023. Table S2. Cure and egg reduction rates in studies of the efficacy of tribendimidine alone and in combination and in comparator treatment arms against intestinal helminths. Background information on traditional medicines evaluated for Dengue, Background information on traditional medicines evaluated for cutaneous leishmaniasis, Table S3. Resources on CURE ID. [file 13071_2023_5909_MOESM1_ESM.docx]

Additional file 1

The pipeline for drugs for control and elimination of Neglected Tropical Diseases: 2. Oral anti-infective drugs and drug combinations for off-label use

Kenneth M. Pfarr^1,2^, Anna K. Krome^1,2,3^, Issraa Al-Obaidi^4^, Hannah Batchelor^4^, Michel Vaillant^5^, Achim Hoerauf^1,2^, Nicholas O. Opoku^6^ and Annette C. Kuesel^7^*

^1^ Institute of Medical Microbiology, Immunology and Parasitology, University Hospital Bonn, Bonn, Germany,

^2^ German Center for Infection Research (DZIF), Partner Site Bonn-Cologne, Bonn, Germany

^3^ Department of Pharmaceutical Technology and Biopharmaceutics, University of Bonn, Bonn, Germany

^4^ Strathclyde Institute of Pharmacy and Biomedical Sciences, University of Strathclyde, UK

^5^ Competence Center for Methodology and Statistics, Luxembourg Institute of Health, Strassen, Grand Duchy of Luxembourg

^6^ Department of Epidemiology and Biostatistics School of Public Health, University of Health and Allied Sciences, Hohoe, Ghana

^7^ UNICEF/UNDP/World Bank/WHO Special Programme for Research and Training in Tropical Diseases (WHO/TDR), World Health Organization, Geneva, Switzerland (retired).

* Correspondence: [kuesela@who.int](mailto:kuesela@who.int), [annette.kuesel@mailbox.org](mailto:annette.kuesel@mailbox.org)

E-mail addresses

KMP: [Kenneth.pfarr@ukbonn.de](mailto:Kenneth.pfarr@ukbonn.de)

AKK: [Krome@uni-bonn.de](mailto:Krome@uni-bonn.de)

IAO: [issraa.al-obaidi@strath.ac.uk](mailto:issraa.al-obaidi@strath.ac.uk)

HB: [hannah.batchelor@strath.ac.uk](mailto:hannah.batchelor@strath.ac.uk)

MV: [Michel.Vaillant@lih.lu](mailto:Michel.Vaillant@lih.lu)

AH: [Achim.Hoerauf@ukbonn.de](mailto:Achim.Hoerauf@ukbonn.de)

NOO: [noopoku@uhas.edu.gh](mailto:noopoku@uhas.edu.gh)

ACK: [kuesela@who.int](mailto:kuesela@who.int) , [annette.kuesel@mailbox.org](mailto:annette.kuesel@mailbox.org)

Table of Contents

[Table S 1: Source registries and last data file import into the WHO Clinical Trials Registry Platform as of 8 February 2023 2](#_Toc130630750)

[Table S 2: Cure and egg reduction rates in studies of the efficacy of tribendimidine alone and in combination and in comparator treatment arms against intestinal helminths 2](#_Toc130630751)

[Background information on traditional medicines evaluated for Dengue 4](#_Toc130630752)

[Background information on traditional medicines evaluated for cutaneous leishmaniasis 6](#_Toc130630753)

[Table S 3: Resources on CURE ID 7](#_Toc130630754)

[References 8](#_Toc130630755)

Table S 1: Source registries and last data file import into the WHO Clinical Trials Registry Platform as of 8 February 2023

| Australian New Zealand Clinical Trials Registry, last data file imported on **30 January 2023** |
| --- |
| Chinese Clinical Trial Registry, last data file imported on **30 January 2023** |
| ClinicalTrials.gov, last data file imported on **30 January 2023** |
| EU Clinical Trials Register (EU-CTR), last data file imported on **30 January 2023** |
| ISRCTN, last data file imported on **30 January 2023** |
| The Netherlands National Trial Register, last data file imported on **30 May 2022** |
| Brazilian Clinical Trials Registry (ReBec), last data file imported on **9 January 2023** |
| Clinical Trials Registry – I OR ndia, last data file imported on **9 January 2023** |
| Clinical Research Information Service - Republic of Korea, last data file imported on **9 January 2023** |
| Cuban Public Registry of Clinical Trials, last data file imported on **9 January 2023** |
| German Clinical Trials Register, last data file imported on **14 November 2022** |
| Iranian Registry of Clinical Trials, last data file imported on **22 November 2022** |
| Japan Registry of Clinical Trials (jRCT), last data file imported on **9 January 2023** |
| Pan African Clinical Trial Registry, last data file imported on **9 January 2023** |
| Sri Lanka Clinical Trials Registry, last data file imported on **9 January 2023** |
| Thai Clinical Trials Registry (TCTR), last data file imported on **11 January 2023** |
| Peruvian Clinical Trials Registry (REPEC), last data file imported on **24 October 2022** |
| Lebanese Clinical Trials Registry (LBCTR), last data file imported on **9 January 2023** |

Table S 2: Cure and egg reduction rates in studies of the efficacy of tribendimidine alone and in combination and in comparator treatment arms against intestinal helminths

| Helminth | Treatment (n analyzed) | CR (%)  (95% CI) | GM ERR (%)  (95% CI) | Additional information |
| --- | --- | --- | --- | --- |
| Hookworm | **Tri Single dose 400 mg** (34) | 63.6 | 77.8 | People’s Republic of China |
| Hookworm | **Tri 200 mg twice**, separated by 6 hours (33) | 47.8 | 65.4 | Randomized, open label study |
| Hookworm | PZQ 75 mg/kg in four doses within 2 days (37) | 18.2 | 32.8 | Kato-Katz 2 samples, 3 smears each, 3 weeks post-Tx |
| Hookworm | Meb single dose 400 mg (30) | 0 | 64.6 | ISRCTN55086560 |
| *A. lumbricoides* | **Tri single dose 400 mg** (14) | 28.6 | 71.1 | [1] |
| *A. lumbricoides* | **Tri 200 mg twice**, separated by 6 hours (12) | 0 | 73.1 |  |
| *A. lumbricoides* | PZQ 75 mg/kg in four doses within 2 days (12) | 16.7 | 66.9 |  |
| *A. lumbricoides* | Meb single dose 400 mg (13) | 7.7 | 79.5 |  |
| *T. trichuris* | **Tri single dose 400 mg** (13) | 23.1 | 77.9 |  |
| *T. trichuris* | **Tri 200 mg twice**, separated by 6 hours (12) | 33.3 | 80.1 |  |
| *T. trichuris* | PZQ 75 mg/kg in four doses within 2 days (13) | 0 | 53.1 |  |
| *T. trichuris* | Meb single dose 400 mg (14) | 0 | 63.8 |  |
|  |  |  |  |  |
| Hookworm | **Tri 400 mg** plus placebo (151) | 53.6  (45.4–61.8) | 96.7  (94.9–97.8) | Tanzania, Côte d’Ivoire |
| Hookworm | **Tri 400 mg** plus IVM 200µg/kg (154) | 84.4  (77.7–89.8) | 99.5  (99.2–99.7) | Randomized, single-blinded, single dose |
| Hookworm | **Tri 400 mg** plus OxP 25mg/kg (148) | 52.0  (43.7–60.3) | 96.5  (94.9–97.6) | Kato-Katz 2 samples, 1 smear each 14-21 post-Tx |
| Hookworm | Alb 400 mg plus OxP 25mg/kg (148) | 48.0  (39.7–56.3) | 96.0  (93.9–97.4) | ISRCTN14373201  [2] |
| *A. lumbricoides* | **Tri 400 mg** plus placebo (151) | 98.6  (92.5–100.0) | >99.99  (>99.99–100.0) |  |
| *A. lumbricoides* | **Tri 400 mg** plus IVM 200µg/kg (154) | 98.7  (92.9–100.0) | >99.99  (>99.99–100.0) |  |
| *A. lumbricoides* | **Tri 400 mg** plus OxP 25mg/kg (148) | (86.0–98.4) | 99.98  (99.93–100.0) |  |
| *A. lumbricoides* | Alb 400 mg plus OxP 25mg/kg (148) | 93.6  (85.7–97.9) | 99.99  (99.96–100.0) |  |
| *T. trichuris* | **Tri 400 mg** plus placebo (151) | 8.2  (3.6–15.6) | 53.1  (32.0–68.3) |  |
| *T. trichuris* | **Tri 400 mg** plus IVM 200µg/kg (154) | 33.7  (24.7–43.6) | 96.4  (94.3–97.8) |  |
| *T. trichuris* | **Tri 400 mg** plus OxP 25mg/kg (148) | 66.3  (55.9–75.7) | 99.5  (99.1–99.7) |  |
| *T. trichuris* | Alb 400 mg plus OxP 25mg/kg (148) | 82.8  (73.9–89.7) | 99.8  (99.7–99.9) |  |
|  |  |  |  |  |
| Hookworm | Moxi 8mg plus Alb 400mg (95) | 76.8%  (66.2–85.4) | 98.9%  (98.0- 99.5) | Tanzania |
| Hookworm | Alb 400mg plus OxP 25 mg/kg (94) | 75.9%  (65.3–84.6) | 98.6%  (97.4- 99.3) | Randomized, single blind, single dose |
| Hookworm | Moxi 8mg plus **Tri [200mg <15 yrs, 400 mg ≥15 yrs]** (55) | 88.2%  (76.1–95.6) | 99.4%  (98.7- 99.8) | Kato-Katz, 2 samples, 2 smears each, 14-21 days post-Tx |
| Hookworm | Moxi 8 mg (51) | 34.0%  (20.8–49.3) | 86.8%  (72.7–93.9) | ISRCTN20398469 |
| *A. lumbricoides* | Moxi 8mg plus Alb 400mg (133) | 96.6%  (91.5–99.1) | >99.9%  (99.98–99.99) | [3] |
| *A. lumbricoides* | Alb 400mg plus OxP 25 mg/kg (129) | 96.6%  (91.4–99.1) | >99.9%  (99.97–99.99) |  |
| *A. lumbricoides* | Moxi 8mg plus **Tri [200mg <15 yrs, 400 mg ≥15 yrs]** (77) | 97.1%  (90.2–99.6) | >99.9%  (99.9–100.0) |  |
| *A. lumbricoides* | Moxi 8 mg (71) | 98.4%  (91.4–99.9) | >99.9%  (99.9–100.0) |  |
| *T. trichuris* | Moxi 8mg plus Alb 400mg (184) | 50.8%  (43.6–57.9) | 98.5%  (98.0–98.9) |  |
| *T. trichuris* | Alb 400mg plus OxP 25 mg/kg (200) | 83.0%  (77.1–87.9) | 99.8%  (99.6–99.9) |  |
| *T. trichuris* | Moxi 8mg plus **Tri [200mg <15 yrs, 400 mg ≥15 yrs]** (119) | 22.7%  (15.5–31.3) | 91.6%  (88.2–93.9) |  |
| *T. trichuris* | Moxi 8 mg (108) | 14.4%  (8.6–22.1) | 83.2%  (77.9–87.6) |  |
|  |  |  |  |  |
| Hookworm | Placebo (34) | 20.6 | 30.6%  (−24.7-64.1) | Côte d’Ivoire |
| Hookworm | **Tri 100 mg** (33) | 21.2 | 65.4%  (24.5–85.9) | Randomized, single blind, single dose |
| Hookworm | **Tri 200 mg** (31) | 38.7 | 82.1%  (58.4–92.5) | Kato-Katz, 2 samples, 2 smears each, 14-21 days post-Tx |
| Hookworm | **Tri 400 mg** (32) | 53.1 | 92.2%  (81.0–97.1) | ISRCTN81391471  [4] |

Alb albendazole, CR cure rate, GM ERR geometric mean based egg reduction rate, IVM ivermectin, Meb mebendazole, Moxi moxidectin, OxP oxantel pamoate, PZQ praziquantel, Tri tribendimidine, Tx treatment

# Background information on traditional medicines evaluated for Dengue

A Phase 2 study to assess the safety and efficacy of AQCH tablets in adults with Dengue fever is ongoing. AQCH is an aqueous extract of the stem of *Cocculus hirsutus* (family: Menispermaceae), a plant with a history of use in traditional medicines. Evaluation of *C. hirsutus* chemical constituents of potential medicinal use dates back to the 1960s [5].The medicinal properties have been linked to several chemical components including phytochemicals like β-sitosterol, trilobine, isotrilobine, syringaresional, protoquercitol, ginnol and related glycosides; cyclopeptide alkaloids and isoquinoline alkaloids found in the stem and Coclaurine, Sinococuline, Magnoflorine, β-Sitosterol, Ginnol and Monomethyl ether of Inositol. ACQH tablets have been formulated to contain not less than: 0.1%w/w magnoflorine; 1.0% sinococulin; 0.1% w/w 20-hydroxyecdysone; 0.05% w/w makisterone-A [6] although pharmacokinetic evaluation of ACQH has focused on the quantification of sinococuline as a marker for ACQH as this was the only agent quantifiable in plasma. The safety of the AQCH tablet formulation has been demonstrated in 60 healthy adult volunteers [7]. The anti-dengue properties of ACQH have been demonstrated in the AG129 mouse model which is an established model for the evaluation of antivirals [8]. Given that this study is conducted by a pharmaceutical company, regulatory registration is possible should AQCH tablets proof to be safe and efficacious treatment of Dengue infections.

*Carica papaya*, commonly known as papaya or pawpaw, has antimicrobial, anthelmintic, antimalarial, antifungal, antiamoebic, hepatoprotective, male and female antifertility, immunomodulatory, and against histaminergic uses and a history of use in traditional medicine, including for dengue fever [9]. The leaves contain phenolic compounds including alkaloids, quercetin and kaempferol and the leaf extract contains antioxidant compounds such as a-tocopherol, ascorbic acid, and flavonoids. Efficacy has been linked to the high flavonoids and phenolic compounds leading to increased platelet activity and the antihemolytic activity of the papaya [10]. The combination of *C. papaya* with doxycycline has been demonstrated to reduce the hospital stay for those with dengue compared to a control or those treated with doxycycline alone [10]. A 2016 systematic review on the efficacy and safety of *C. papaya* leaf extract in dengue [11], concluded that treatment with *C. papaya* leaf extract results in an increased platelet count and a decrease in hospitalisation days. Possible mechanism of action have recently been reviewed [12].

Ganghuo Kanggan decoction (GHKGD) is a multicomponent traditional Chinese medicine. As for many other traditional medicines, research into the mechanism or mechanisms of action, and the ingredients responsible is ongoing [13].

*Eupatorium perfoliatum* is popularly known as “bone set” as it brings relief in severe bone and joint pains in fever. Bioactive components of *E. perfoliatum* include quercetin, caffeic acid and eupafolin, all of which are known to have antiviral potential. *E. perfoliatum* extracts were shown to have anti-viral activity against influenza A virus in a cell based assay [14]and against DENV infection in the HepG2 cell line [15]. A retrospective cohort study on the use of E. perfoliatum 200C on the prevention of dengue hemorrhagic fever showed that the use of this remedy had a significant effect on the prevention of hemorrhagic fever (<https://he01.tci-thaijo.org/index.php/muhed/article/view/187937>).

# Background information on traditional medicines evaluated for cutaneous leishmaniasis

Several studies in clinical trial registries assess the efficacy of traditional/phytomedicines, including studies of plant extracts whose antileishmanial activity or activity against other infectious agents has been demonstrated in *in vitro* or *in vivo* studies.

Extracts of *Perovskia abrotanoides* contain monoterpenes and sequiterpene, e.g., 1,8-cineole, myrecene, pinene, camphor, caryophyllene, humulene, camphene, bisabolol, abietane diterpenoids and 11-O and 12-O-acetylcarnosic acids have antimicrobial activity with MICs of 1.2 mg/mL [16]. Together with *Aloe vera, Nigella sativa,* propolis, lavender and olive oil, as a topical preparation, *P. abrotanoides* significatnly decreased lesion size and parasite load equivalent to glucantime in BALB/c mice infected with *Leishmania major [17]*. A study comparing *P. abrotanoides* extract (10% topical Brazambel QD) versus glucantime (intralesional infection twice a week for 8 weeks) has completed recruitment [IRCT20150721023282N19].

Methanolic extracts of *Berberis vulgaris* contain the isoquinoline alkaloids berbamine, palmatine and berberine, which have been shown to be antimicrobial and antifungal. IC_50_ ranges against promastigotes of *L*. *infantum* and *L. tropica* were 13.2-16.1 µgmL methanolic extract, 2.7-2.9 µg/mL berberine. IC_50_ ranges against amastigotes of *L*. *infantum* and *L. tropica* were 32-39 µg/mL methanolic extract, 3.9-4.7 µg/mL berberine [18, 19]. In both species, the methanolic extract inhibited infection of macrophages by >52%, while berbine inhibited infection of macrophages by >79%. A study comparing topical *B. vulgaris* methanolic extract (TID for 8 weeks) as monotherapy and in combination with glucantime (intralesional injection weekly for 8 weeks) with glucantime alone completed recruitment in 2016 [IRCT2015060122512N1].

The green tea catechin epigallocatechin gallate was shown to have *in vitro* activity against *L. amazonensis* and *L. braziliensis* via mitochondrial damage and reactive oxygen species, respectively [20, 21]. *L. amazonensis* promastigote proliferation was inhibited in a dose dependent manner with an IC_50_ of 0.063 mM after 120 hours of incubation. Against *L. braziliensis,* epigallocatechin inhibited viability by 80.7% with an IC_50_ of 278 µM. BALB/c mice infected with *L. braziliensis* had a significant reduction in lesion size and parasitemia within one week of 100 mg/kg/day oral epigallocatechin [21]. A topical ointment of epigallocatechin also significantly reduced lesion size and parasite burden in infected mice, equivalent to glucantime [22]. As per the clinical trial registry record [IRCT20180526039848N2] a study evaluating a 2% and 5% topical ointment in combination with glucantime versus glucantime standard therapy has completed recruitment.

*Zataria multiflora* extracts contain thymol, carvacrol, and *p*-cymene. Both the essential oil and methanolic extracts have antileishmanial activity against *L. tropica* promastigotes (IC_50_ 3.2 µL/mL and 9.8 µL/mL, respectively) and amastigotes (IC_50_ 8.3 µL/mL and 34.6 µL/mL, respectively) [23]. As per the ICTR records, three studies have completed recruitment: (1) A study comparing topical gel containing 0.5 g thyme BID plus glucantime standard therapy vs glucantime standard therapy [IRCT20211211053349N1]; (2) A study comparing an herbal paste containing *Z. multiflora* and *Lowsonia inermis* (henna), which inhibits *L. major* promastigote growth with an IC_50_ 1.25 mg/mL after 72 hours [24], and opium applied daily to glucantime standard therapy [IRCT20150105020570N3]; (3) a study evaluating the effect of the *Z. multiflora* component thymol, which has antimicrobial and antifungal activity [25] as 5, 10, and 15% chitosan gels compared to that of glucantime standard therapy [IRCT20180526039848N1].

Methanolic extract of *Chelidonium majus*, containing the alkaloids protoberberines, allocryptopine, protopine, sanguinarine, chelerythrine and chelidonine, has been shown to have antileishmanial activity against promastigotes (IC_50_ of 0.92 µg/mL) and *L. major* intra-macrophage amastigotes (90 µg/mL resulting in cytotoxicity of 50% and 59% after 24 and 48 hours, respectively) [26]. A topical cream containing 0.5 g *C. majus* together with 0.5 g *Hyoscyamus niger* and 2 g propolis, which have repair and anti-infective synergy, respectively [27, 28], is being compared to the use of amphotericin B cream; both interventions given once daily for 7 days. The study has completed recruitment [IRCT20200516047462N5].

Methanolic and ethanolic extract of *Casuarina equisetifolia* has some activity, inhibiting urease, against *Helicobacter pylori* (MICs = 32–256 µg/mL against 34 clinical isolates [29]. Aqueous and ethanolic extracts of *Thespesia populnea* root were active against Gram-positive and Gram-negative bacteria with MICs of 75-500 µg/mL against *Staphylococcus aureus, Escherichia coli, Pseudomonas aeruginosa, Bacillus subtilis* and *Klebsiella aerogenes* , and 10-250 µg/mL when using the ethanolic extract [30]. The potential antileishmanial activity of two topical formulations containing 5% *C. equisetifolia* L. and *Thespesia populnea* L*.* plant extract in BALB/c mice has been investigated in the context of a completed clinical trial [NCT04841239].

The phenolic pigment gossypol from *Gossypium herbaceum* has been shown to prevent recovery of viable virus particles from enveloped viruses: HIV-1, influence, parainfluenza [31]. Standardized formulations of ethanolic topical preparations were being developed to evaluate the effect on cutaneous leishmaniasis in a blinded RCT [IRCT20220131053899N1] which has completed recruitment.

Table S 3: Resources on CURE ID

| About Cure ID | <https://cure.ncats.io/about> |
| --- | --- |
| Cure ID app | <https://cure.ncats.io/home> |
| Contact information | [curesupport@mail.nih.gov](mailto:curesupport@mail.nih.gov) |
| CURE Drug Repurposing Collaboratory | <https://c-path.org/programs/cdrc/> |
| CURE ID App Lets Clinicians Report Novel Uses of Existing Drugs | <https://www.fda.gov/drugs/science-and-research-drugs/cure-id-app-lets-clinicians-report-novel-uses-existing-drugs> |
| CURE ID Moves to Automated Data Collection in Light of COVID Pandemic | <https://www.fda.gov/drugs/news-events-human-drugs/cure-id-moves-automated-data-collection-light-covid-pandemic> |

# References

1. Xu LL, Jiang B, Duan JH, Zhuang SF, Liu YC, Zhu SQ, et al. Efficacy and safety of praziquantel, tribendimidine and mebendazole in patients with co-infection of *Clonorchis sinensis* and other helminths. PLoS Negl Trop Dis. 2014;8 8:e3046; doi: 10.1371/journal.pntd.0003046. <https://www.ncbi.nlm.nih.gov/pubmed/25122121>

<https://www.ncbi.nlm.nih.gov/pmc/articles/PMC4133228/pdf/pntd.0003046.pdf>.

2. Moser W, Coulibaly JT, Ali SM, Ame SM, Amour AK, Yapi RB, et al. Efficacy and safety of tribendimidine, tribendimidine plus ivermectin, tribendimidine plus oxantel pamoate, and albendazole plus oxantel pamoate against hookworm and concomitant soil-transmitted helminth infections in Tanzania and Cote d'Ivoire: a randomised, controlled, single-blinded, non-inferiority trial. Lancet Infect Dis. 2017;17 11:1162-71; doi: 10.1016/S1473-3099(17)30487-5. <https://www.ncbi.nlm.nih.gov/pubmed/28864027>.

3. Barda B, Ame SM, Ali SM, Albonico M, Puchkov M, Huwyler J, et al. Efficacy and tolerability of moxidectin alone and in co-administration with albendazole and tribendimidine versus albendazole plus oxantel pamoate against *Trichuris trichiura* infections: a randomised, non-inferiority, single-blind trial. Lancet Infect Dis. 2018;18 8:864-73; doi: 10.1016/S1473-3099(18)30233-0. <https://www.ncbi.nlm.nih.gov/pubmed/29858149>.

4. Coulibaly JT, Hiroshige N, N'Gbesso YK, Hattendorf J, Keiser J. Efficacy and safety of ascending dosages of tribendimidine against hookworm infections in children: A randomized controlled trial. Clin Infect Dis. 2019;69 5:845-52; doi: 10.1093/cid/ciy999. <https://www.ncbi.nlm.nih.gov/pubmed/30496350>

.

5. Logesh R, Das N, Adhikari-Devkota A, Devkota HP. Cocculus hirsutus (L.) W.Theob. (Menispermaceae): A Review on Traditional Uses, Phytochemistry and Pharmacological Activities. Medicines (Basel). 2020;7 11; doi: medicines7110069 [pii];10.3390/medicines7110069 [doi]. <http://www.ncbi.nlm.nih.gov/pubmed/33182572>.

6. Government of India (Central Drugs Standard Control Organization New Drugs Division of the Directorate General of Health Services), Dehli. 2021. <https://cdsco.gov.in/opencms/resources/UploadCDSCOWeb/2018/UploadCTApprovals/22%20Sun%20AQCH%20CT-06.pdf>.

7. Dar SK, Kumar S, Maiti S, Dhawan S, Joglekar S, Arora U, et al. Clinical safety and pharmacokinetic evaluation of aqueous extract of Cocculus hirsutus, an anti-viral phytopharmacetical drug as a potential for the treatment of dengue and COVID-19. Heliyon. 2022;8 5:e09416; doi: 10.1016/j.heliyon.2022.e09416 [doi];S2405-8440(22)00704-6 [pii]. <http://www.ncbi.nlm.nih.gov/pubmed/35582330>.

8. Shukla R, Rajpoot RK, Poddar A, Ahuja R, Beesetti H, Shanmugam RK, et al. Cocculus hirsutus-Derived Phytopharmaceutical Drug Has Potent Anti-dengue Activity. Front Microbiol. 2021;12:746110; doi: 10.3389/fmicb.2021.746110 [doi]. <http://www.ncbi.nlm.nih.gov/pubmed/34912307>.

9. Sarker MMR, Khan F, Mohamed IN. Dengue Fever: Therapeutic Potential of Carica papaya L. Leaves. Frontiers in Pharmacology. 2021;12; doi: 10.3389/fphar.2021.610912. <https://www.frontiersin.org/articles/10.3389/fphar.2021.610912>.

10. Pambhar V, Mathur N, Mehta A, Mathur M, Kumawat DC, Mangalia R, et al. Effect of doxycycline and doxycycline with carica papaya on thrombocytopenia and leucopenia in acute dengue fever patients. Journal of Family Medicine and Primary Care. 2022;11 6:3270-5; doi: 10.4103/jfmpc.jfmpc_53_22. <https://journals.lww.com/jfmpc/Fulltext/2022/06000/Effect_of_doxycycline_and_doxycycline_with_carica.161.aspx>.

11. Charan J, Saxena D, Goyal JP, Yasobant S. Efficacy and safety of Carica papaya leaf extract in the dengue: A systematic review and meta-analysis. Int J Appl Basic Med Res. 2016;6 4:249-54; doi: 10.4103/2229-516X.192596. <https://www.ncbi.nlm.nih.gov/pubmed/27857891>.

12. Bok ZK, Balakrishnan M, Jong YX, Kong YR, Khaw KY, Ong YS. The plausible mechanisms of action of *Carica papaya* on Dengue infection: A comprehensive review. Progress in Drug Discovery & Biomedical Science. 2020;3 1:a0000097; doi: <https://doi.org/10.3687/pddbs.a0000097>. blob:<https://journals.hh-publisher.com/3c35cb3d-8aa6-4cdf-a5d6-99b0afcf6712>.

13. Lai Y, Zhang Q, Long H, Han T, Li G, Zhan S, et al. Ganghuo Kanggan Decoction in Influenza: Integrating Network Pharmacology and In Vivo Pharmacological Evaluation. Frontiers in Pharmacology. 2020;11; doi: 10.3389/fphar.2020.607027. <https://www.frontiersin.org/articles/10.3389/fphar.2020.607027>.

14. Derksen A, Kühn J, Hafezi W, Sendker J, Ehrhardt C, Ludwig S, et al. Antiviral activity of hydroalcoholic extract from Eupatorium perfoliatum L. against the attachment of influenza A virus. Journal of Ethnopharmacology. 2016;188:144-52; doi: <https://doi.org/10.1016/j.jep.2016.05.016>. <https://www.sciencedirect.com/science/article/pii/S0378874116302793>.

15. Sinha M, Chakraborty U, Kool A, Chakravarti M, Das S, Ghosh S, et al. In-vitro antiviral action of Eupatorium perfoliatum against dengue virus infection: Modulation of mTOR signaling and autophagy. Journal of Ethnopharmacology. 2022;282:114627; doi: <https://doi.org/10.1016/j.jep.2021.114627>. <https://www.sciencedirect.com/science/article/pii/S0378874121008564>.

16. Abedini A, Roumy V, Mahieux S, Gohari A, Farimani MM, Riviere C, et al. Antimicrobial activity of selected Iranian medicinal plants against a broad spectrum of pathogenic and drug multiresistant micro-organisms. Lett Appl Microbiol. 2014;59 4:412-21; doi: 10.1111/lam.12294. <https://www.ncbi.nlm.nih.gov/pubmed/24888993>.

17. Saberi R, Zadeh AG, Afshar MJA, Fakhar M, Keighobadi M, Mohtasebi S, et al. In vivo anti-leishmanial activity of concocted herbal topical preparation against Leishmania major (MRHO/IR/75/ER). Ann Parasitol. 2021;67 3:483-8; doi: 10.17420/ap6703.361. <https://www.ncbi.nlm.nih.gov/pubmed/34953123>.

18. Mahmoudvand H, Sharififar F, Rahmat MS, Tavakoli R, Dezaki ES, Jahanbakhsh S, et al. Evaluation of antileishmanial activity and cytotoxicity of the extracts of Berberis vulgaris and Nigella sativa against Leishmania tropica. J Vector Borne Dis. 2014;51 4:294-9. <https://www.ncbi.nlm.nih.gov/pubmed/25540961>.

19. Mahmoudvand H, Sharififar F, Sharifi I, Ezatpour B, Fasihi Harandi M, Makki MS, et al. In Vitro Inhibitory Effect of Berberis vulgaris (Berberidaceae) and Its Main Component, Berberine against Different Leishmania Species. Iran J Parasitol. 2014;9 1:28-36. <https://www.ncbi.nlm.nih.gov/pubmed/25642257>.

20. Inacio JD, Canto-Cavalheiro MM, Menna-Barreto RF, Almeida-Amaral EE. Mitochondrial damage contribute to epigallocatechin-3-gallate induced death in Leishmania amazonensis. Exp Parasitol. 2012;132 2:151-5; doi: 10.1016/j.exppara.2012.06.008. <https://www.ncbi.nlm.nih.gov/pubmed/22735546>.

21. Inacio JD, Gervazoni L, Canto-Cavalheiro MM, Almeida-Amaral EE. The effect of (-)-epigallocatechin 3-O--gallate in vitro and in vivo in Leishmania braziliensis: involvement of reactive oxygen species as a mechanism of action. PLoS Negl Trop Dis. 2014;8 8:e3093; doi: 10.1371/journal.pntd.0003093. <https://www.ncbi.nlm.nih.gov/pubmed/25144225>.

22. Sosa AM, Moya Alvarez A, Bracamonte E, Korenaga M, Marco JD, Barroso PA. Efficacy of Topical Treatment with (-)-Epigallocatechin Gallate, A Green Tea Catechin, in Mice with Cutaneous Leishmaniasis. Molecules. 2020;25 7; doi: 10.3390/molecules25071741. <https://www.ncbi.nlm.nih.gov/pubmed/32290128>.

23. Saedi Dezaki E, Mahmoudvand H, Sharififar F, Fallahi S, Monzote L, Ezatkhah F. Chemical composition along with anti-leishmanial and cytotoxic activity of Zataria multiflora. Pharm Biol. 2016;54 5:752-8; doi: 10.3109/13880209.2015.1079223. <https://www.ncbi.nlm.nih.gov/pubmed/26449681>.

24. Motazedian MH, Mikaeili F, Mohebali M, Miri R, Habibi P, Kamarloie S. The antileishmanial effects of Lowsonia inermis and Cedrus libani on Leishmania major promastigotes: an in vitro study. J Parasit Dis. 2017;41 2:375-9; doi: 10.1007/s12639-016-0809-y. <https://www.ncbi.nlm.nih.gov/pubmed/28615844>.

25. Nagoor Meeran MF, Javed H, Al Taee H, Azimullah S, Ojha SK. Pharmacological Properties and Molecular Mechanisms of Thymol: Prospects for Its Therapeutic Potential and Pharmaceutical Development. Front Pharmacol. 2017;8:380; doi: 10.3389/fphar.2017.00380. <https://www.ncbi.nlm.nih.gov/pubmed/28694777>.

26. Madjeed Haddao K, Dawood Saleem H, Hameed NM, Mahdi Rheima A, Alkhafaje WK, Salaam Abood E, et al. Investigation of in vitro Cytotoxicity of Chelidonium majus against Leishmania major. Arch Razi Inst. 2022;77 3:1211-4; doi: 10.22092/ARI.2022.358758.2301. <https://www.ncbi.nlm.nih.gov/pubmed/36618311>.

27. Kosari M, Noureddini M, Khamechi SP, Najafi A, Ghaderi A, Sehat M, et al. The effect of propolis plus Hyoscyamus niger L. methanolic extract on clinical symptoms in patients with acute respiratory syndrome suspected to COVID-19: A clinical trial. Phytother Res. 2021;35 7:4000-6; doi: 10.1002/ptr.7116. <https://www.ncbi.nlm.nih.gov/pubmed/33860587>.

28. Salatino A. Perspectives for Uses of Propolis in Therapy against Infectious Diseases. Molecules. 2022;27 14; doi: 10.3390/molecules27144594. <https://www.ncbi.nlm.nih.gov/pubmed/35889466>.

29. Amin M, Anwar F, Naz F, Mehmood T, Saari N. Anti-Helicobacter pylori and urease inhibition activities of some traditional medicinal plants. Molecules. 2013;18 2:2135-49; doi: 10.3390/molecules18022135. <https://www.ncbi.nlm.nih.gov/pubmed/23434867>.

30. Senthil-Rajan D, Rajkumar M, Srinivasan R, Kumarappan C, Arunkumar K, Senthilkumar KL, et al. Investigation on antimicrobial activity of root extracts of Thespesia populnea Linn. Trop Biomed. 2013;30 4:570-8. <https://www.ncbi.nlm.nih.gov/pubmed/24522124>.

31. Perera W, Liyanage JA, Dissanayake KGC, Gunathilaka H, Weerakoon W, Wanigasekara DN, et al. Antiviral Potential of Selected Medicinal Herbs and Their Isolated Natural Products. Biomed Res Int. 2021;2021:7872406; doi: 10.1155/2021/7872406. <https://www.ncbi.nlm.nih.gov/pubmed/34926691>.
